# In Vitro Modulatory Effect of Stimulant Beverages on the Growth Kinetics of Intestinal Bacteria Associated with Colorectal Cancer

**DOI:** 10.3390/foods15193532

**Published:** 2026-10-03

**Authors:** Tomas Kudera, Diana Karina Baigts-Allende, Barbora Fiserova, Ladislav Kokoska

**Affiliations:** 1 Department of Crop Sciences and Agroforestry, Faculty of Tropical AgriSciences, Czech University of Life Sciences Prague, Kamycka 129, Praha-Suchdol, 165 00 Prague, Czech Republic; kuderat@ftz.czu.cz (T.K.); fiserovab@ftz.czu.cz (B.F.); 2 Institute for Obesity Research and School of Medicine and Health Sciences, Tecnologico de Monterrey, Monterrey 64849, Mexico; baigts.diana@tec.mx

**Keywords:** oncogenic bacteria, hot drinks, growth kinetics

## Abstract

Colorectal cancer is increasingly linked to diet-induced gut microbiota modulations, whereas the role of oncogenic bacteria is discussed. This study investigated the in vitro growth-modulatory effects of hot-water extracts of six stimulant beverages, namely *Aspalathus linearis* (rooibos), *Coffea arabica*, *Coffea canephora* (coffee), *Camellia sinensis* var. *assamica*, *C. sinensis* var. *sinensis* (tea) and *Ilex paraguariensis* (yerba mate) against six colorectal cancer-associated bacteria. Although no minimum inhibitory concentration (MIC) was detected (MIC > 512 µg/mL), growth kinetics analysis revealed modulations in the case of several strains. At the concentration 512 µg/mL, *C. sinensis* var. *assamica* and *I. paraguariensis* modulated the growth (41–56%) and prolonged the lag phase (61–128 min) of *Clostridium septicum*. The same concentration of both extracts modulated *Fusobacterium necrophorum* (26–27%), whereas *I. paraguariensis* extended the lag phase by 72 and 21 min in the cases of *Peptostreptococcus anaerobius* and *Escherichia coli*, respectively. Both *C. sinensis* varieties also reduced the growth of *Streptococcus bovis* (49–60%), while some treatments stimulated the growth of *E. coli*. These findings demonstrate in vitro growth modulation of selected colorectal cancer-associated bacteria, especially *C. sinensis* and *I. paraguariensis*, and warrant validation using multiple strains, independently replicated assays, chemically characterized extracts, and more physiologically relevant microbial models.

## 1. Introduction

In 2020, colorectal cancer was the third-most-diagnosed malignancy in the world following breast and lung cancers, and the second leading cause of cancer death after the lung cancer [1]. Despite the decline in mortality and incidence rates for adults over 50 in recent decades, the overall number of cases has increased, particularly among individuals aged 20 to 49 [2]. In fact, the global burden of colorectal cancer is expected to rise by 60% to more than 2.2 million new cases and 1.1 million deaths by 2030 [3]. Excluding the genetic predisposition risks [4], over half of all cases are linked to lifestyle-related factors that contribute to the development of this malignancy. Apart from the lack of physical activity, stress, and other unfavorable habits, diet has been suggested as the major factor influencing the disease progression [5]. Multiple studies have recently indicated a close relationship between diet and the occurrence, development, and prognosis of various cancers. Because the digestive system directly interacts with food, dietary interventions may be particularly relevant to the prevention and management of gastrointestinal malignancies [6]. It is well known that diet can directly influence colorectal carcinogenesis through exposure to carcinogenic compounds and the metabolic effects of nutrients [7]. However, growing attention has recently been directed toward the indirect effect of food on carcinogenesis mediated through diet-induced alterations in the gastrointestinal microbiota [8].

The human gastrointestinal tract contains an enormous and complex community of commensal microorganisms that provides crucial functions contributing to the host’s health such as nutrient uptake, metabolism of xenobiotics, and to the development of human immune system [9]. The bacterial divisions *Actinobacteria*, *Bacteroides*, *Cyanobacteria*, *Firmicutes*, *Fusobacteria*, *Proteobacteria*, and *Verrucomicrobia* are the main representatives, whereas their taxonomic diversity and compositional balance are crucial for the maintenance of homeostasis and the symbiotic relationship with the gastrointestinal system of the host [10]. Nevertheless, some species are recognized for their probiotic functions, supporting gut equilibrium, while others are known as facultative or obligate gastrointestinal pathogens, which impair this balance and cause dysbiosis. The former group includes beneficial probiotic bacteria, particularly strains found in numerous fermented food products (e.g., *Bifidobacterium* and *Lactobacillus* spp.) [11], whereas the latter group includes enteric bacteria and foodborne pathogens like *Aeromonas* spp., *Campylobacter* spp., *Clostridium difficile*, *Escherichia coli*, *Salmonella* spp., and *Shigella* spp. [12]. The association between dysbiotic changes in gut microbiome composition and inflammatory processes leading to intestinal carcinogenesis has been increasingly discussed [13,14]. There is rising evidence that bacteria are a significant part of the tumor microenvironment that can contribute to colorectal cancer metastasis by signaling through metabolites, promoting epithelial–mesenchymal transition, creating an immunosuppressive microenvironment, and disrupting the gut–vascular barrier [15]. In addition to the standard enteric pathogens, specific bacterial species significant in colorectal cancer evolution have been identified, especially *Fusobacterium nucleatum*, colibactin-producing *E. coli*, and toxigenic *Bacteroides fragilis* [16]. In the case of *F. nucleatum*, the FadA antigen is a ligand for E-cadherin on intestinal epithelial cells that activates the β-catenin signaling pathway, thereby promoting uncontrolled cell growth, acquisition of a stem cell-like phenotype of epithelia and loss of cell polarity. Regarding *E. coli* oncogenic strains, the genomic polyketide synthase island encodes the genotoxin colibactin that can induce DNA damage and mutations in epithelial cells [17]. Other bacteria with a suggested oncogenic role in the human intestines are *Alistipes* spp., *Bilophila wadsworthia*, *Clostridium septicum*, *Enterococcus faecalis*, *Eubacterium rectale*, *Fusobacterium necrophorum*, *Parvimonas* spp., *Peptostreptococcus anaerobius*, *Streptococcus bovis*, *Streptococcus gallolyticus* [14,17,18,19,20,21]. On the other hand, anti-inflammatory and cancer-preventive effects have been discussed in the cases of *Akkermansia muciniphila* and *Faecalibacterium prausnitzii* [22,23]. The abundance and diversity of intestinal bacteria present in the gut microbiota can be greatly affected by diet, not only through fermentable substrates and live microorganisms present in some foods, but also through bioactive compounds that can selectively promote or inhibit bacterial growth [24,25,26,27,28,29,30].

Stimulant beverages are infusions, decoctions and macerates from plants or plant parts that are cultivated for their chemical constituents, which have a stimulating effect on the central nervous system. Apart from the most widely consumed infusions of *Camellia sinensis* leaves (tea) and seeds of *Coffea arabica* (arabica coffee) and *Coffea canephora* (robusta coffee), other stimulant beverages that have been in global popularity include the leaf infusions of *Aspalathus linearis* (rooibos) and *Ilex paraguariensis* (yerba mate) [30,31,32]. High consumption rates of stimulant beverages, particularly tea and coffee, make them significant sources of bioactive compounds, such as polyphenols and antioxidants, which are known for their health-promoting properties [33,34]. Human evidence linking stimulant beverage consumption and their constituents to colorectal cancer remains limited and heterogeneous, with most studies focusing on coffee and tea. Several intervention studies reported reduced colorectal adenoma recurrence after green tea extract supplementation [35,36,37,38], whereas meta-analysis of randomized controlled trials indicated that epigallocatechin gallate (EGCG) may be the responsible compound [39]. In contrast, other trials found no overall benefit of the tea consumption or effects limited to specific subgroups [40,41]. In the case of *C. arabica*, the prospective observational analysis of patients with advanced or metastatic colorectal cancer showed that greater coffee consumption was associated with lower risks of disease progression and death [42]. Although this clinical evidence remains inconclusive, stimulant beverages and their constituents have also been shown to influence the composition of gastrointestinal microbiota, including bacterial groups associated with colorectal carcinogenesis. For example, Yuan et al. [43] found that fecal samples of volunteers drinking green tea had selectively decreased the proinflammatory *Fusobacterium* genus. In a mice colitis model, the study of Olate-Briones et al. [44] showed that oral administration of *I. paraguariensis* modulated the gut microbiota, reducing the *E. rectale*, *C. coccoides* and Enterobacteriaceae groups.

These observations indicate that stimulant beverages can influence gut bacterial populations involved in carcinogenesis, although their potential role in colorectal cancer prevention has not yet been clearly established from a clinical perspective. One potential mechanism may involve antimicrobial and growth-modulatory properties. The antimicrobial properties of stimulant beverage-derived products have previously been reported in a number of studies. Green tea extracts and their constituent EGCG have shown inhibitory activity against several pathogenic bacteria, including *E. coli*, *S. aureus*, *P. aeruginosa*, *F. nucleatum*, and *S. mutans* [45,46,47]. Growth-inhibitory activity has likewise been reported for coffee extracts against *S. aureus*, *Vibrio cholerae*, *S. mutans*, and *F. nucleatum* [48,49,50,51], for *I. paraguariensis* against *E. coli*, *Salmonella* spp., enterococci, and *Listeria monocytogenes* [52,53,54,55,56], and for *A. linearis* against *E. coli*, *E. faecalis*, and *Bacillus cereus* [57]. While previously reported MICs are generally higher than those typical for antibiotics and the in vitro antimicrobial effect should be classified as moderate or weak when compared with other plant-derived products [58], the role of stimulant beverages in gut microbiota modulation through the potential suppression of colorectal cancer-associated bacteria may still be significant because of their relatively high, frequent and continuous intake by the consumers [59]. Accordingly, measuring bacterial growth kinetics at sub-inhibitory concentrations is well suited to this question, because it can reveal time-dependent effects on bacterial adaptation, growth and death that a single minimum inhibitory concentration (MIC) value cannot capture. Such measurements may therefore identify effects of beverage constituents that remain relevant under sustained exposure even when the MICs indicate only modest antimicrobial activity [60].

Although the in vitro antimicrobial activity of the above-mentioned stimulant beverages has previously been studied, the data on their inhibitory actions against colorectal cancer-related bacteria are very limited, particularly with respect to their influence on bacterial growth kinetics at sub-MICs. Due to that, in this study, in vitro growth-modulatory properties of extracts (freeze-dried hot-water infusion residues dissolved in DMSO) of *A. linearis*, *C. sinensis* (var. *sinensis* and var. *assamica*), *C. arabica*, *C. canephora*, and *I. paraguariensis* were tested against several colorectal cancer-related bacterial strains with special focus on their impact on microbial growth kinetics.

## 2. Results

**Figure 1:**
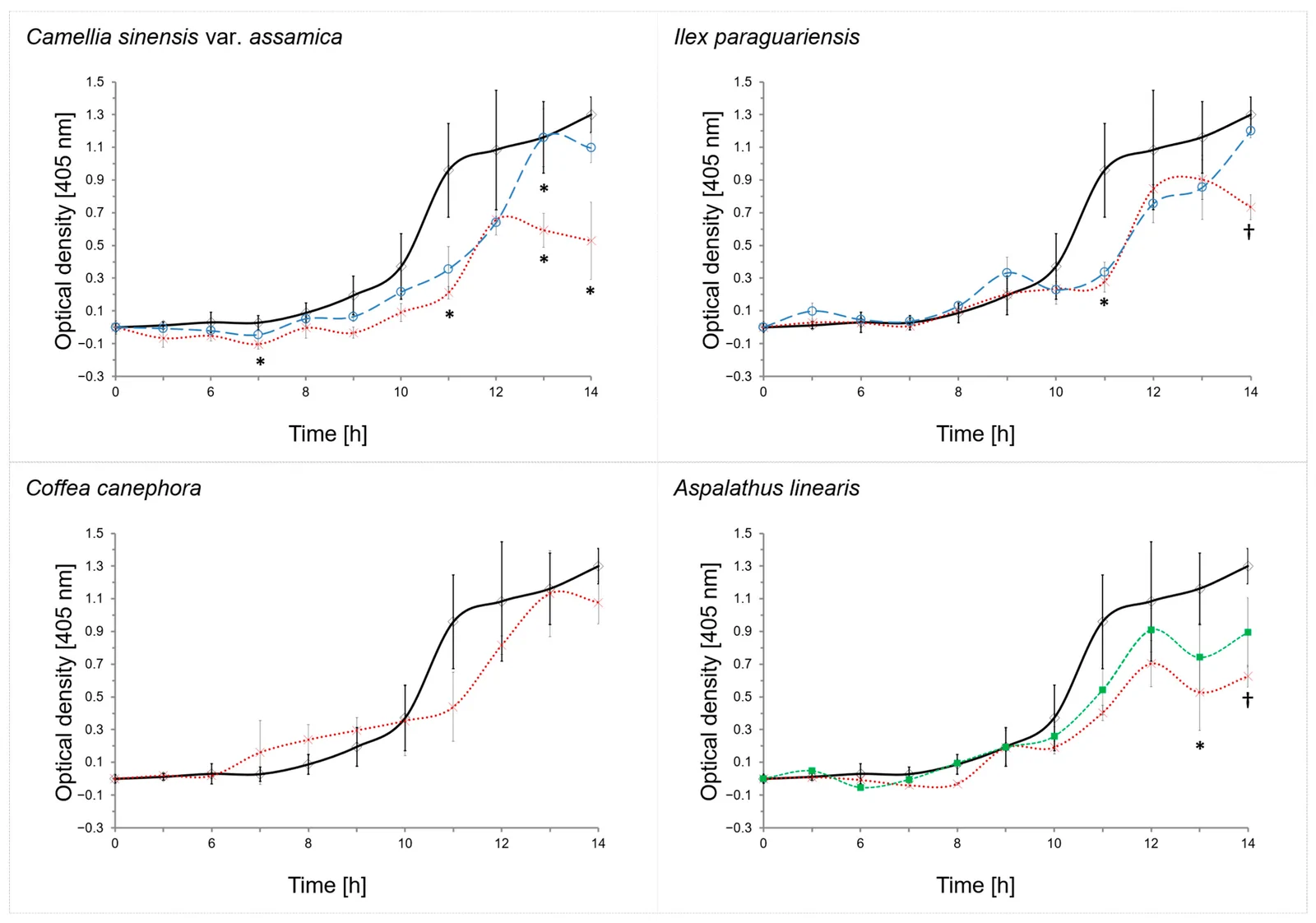
The effect of hot-water extracts from stimulant beverages on the growth kinetics of *Clostridium septicum* ATCC 12464. Growth control (**—**◇**—**), 512 µg/mL (···×···), 256 µg/mL (– –○– –), 128 µg/mL (---■---); * *p* < 0.05 vs. growth control (Welch’s *t*-test; unadjusted); † adjusted *p* < 0.05 (Bonferroni correction). Data are mean ± standard deviation, *n* = 3 technical replicates.

**Figure 2:**
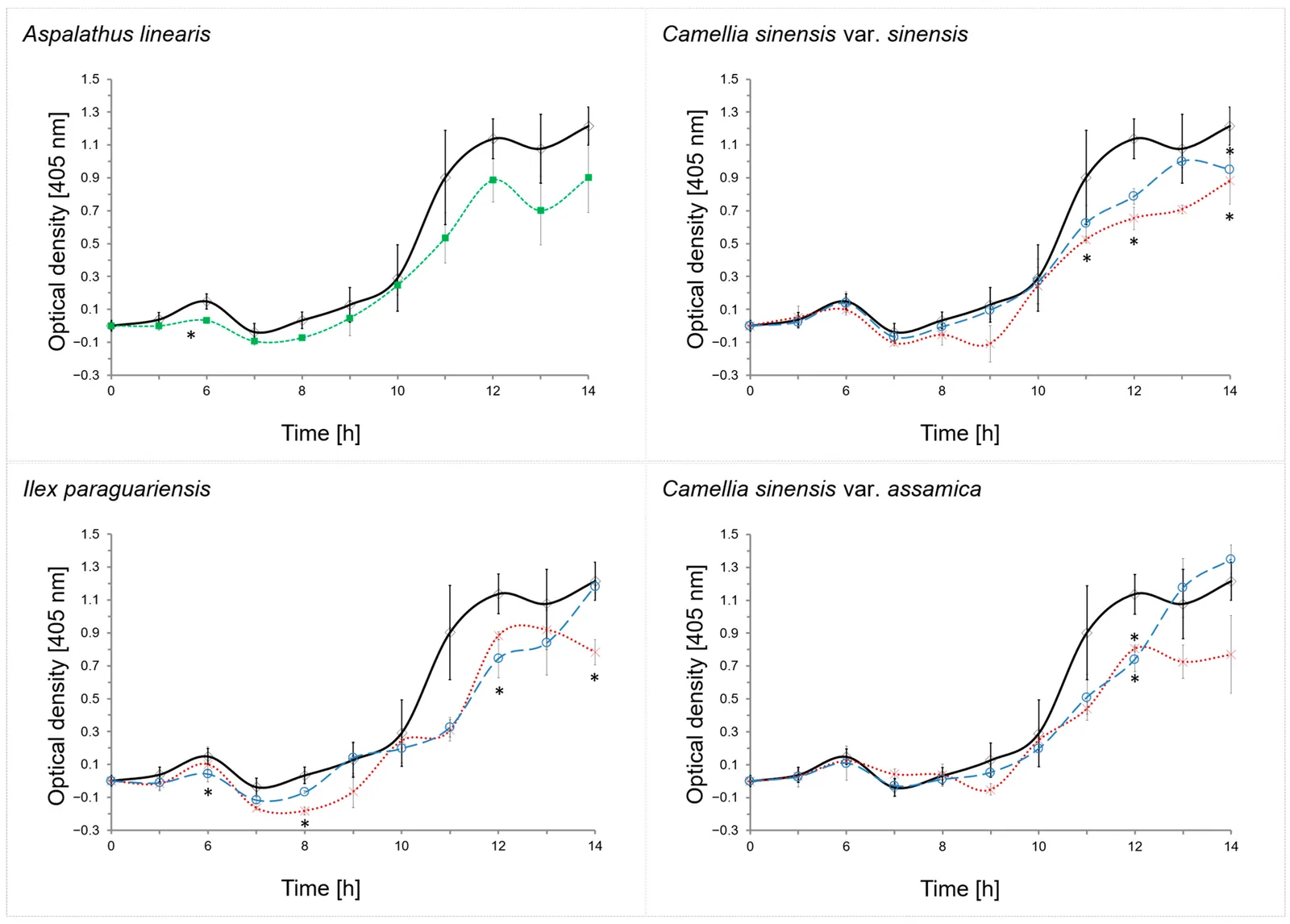
The effect of hot-water extracts from stimulant beverages on the growth kinetics of *Peptostreptococcus anaerobius* CCM 3790. Growth control (**—**◇**—**), 512 µg/mL (···×···), 256 µg/mL (– –○– –), 128 µg/mL (---■---); * *p* < 0.05 vs. growth control (Welch’s *t*-test; unadjusted). Data are mean ± standard deviation, *n* = 3 technical replicates.

**Figure 3:**
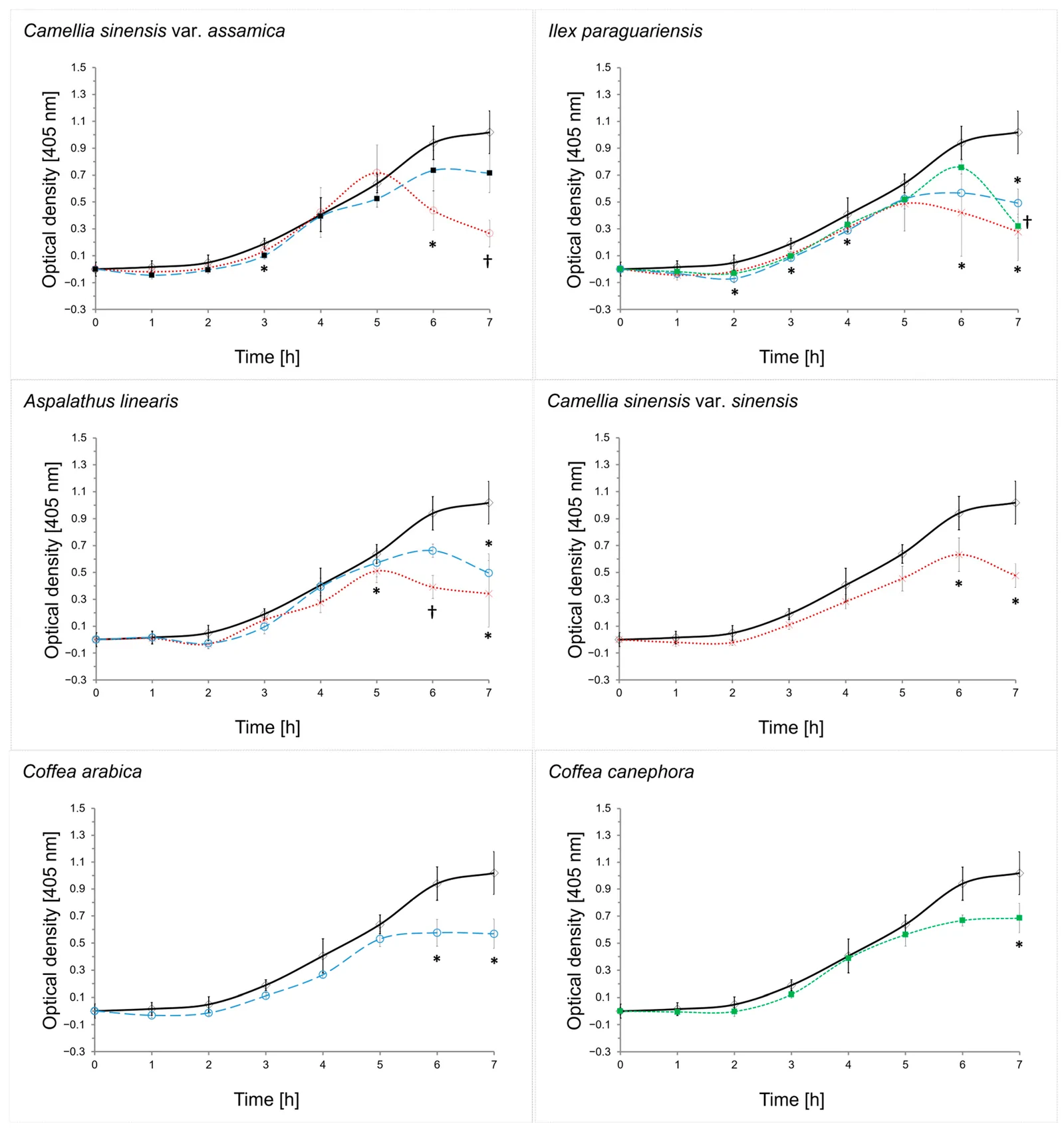
The effect of hot-water extracts from stimulant beverages on the growth kinetics of *Fusobacterium necrophorum* CCM 5981. Growth control (**—**◇**—**), 512 µg/mL (···×···), 256 µg/mL (– –○– –), 128 µg/mL (---■---); * *p* < 0.05 vs. growth control (Welch’s *t*-test; unadjusted); † adjusted *p* < 0.05 (Bonferroni correction). Data are mean ± standard deviation, *n* = 3 technical replicates.

**Figure 4:**
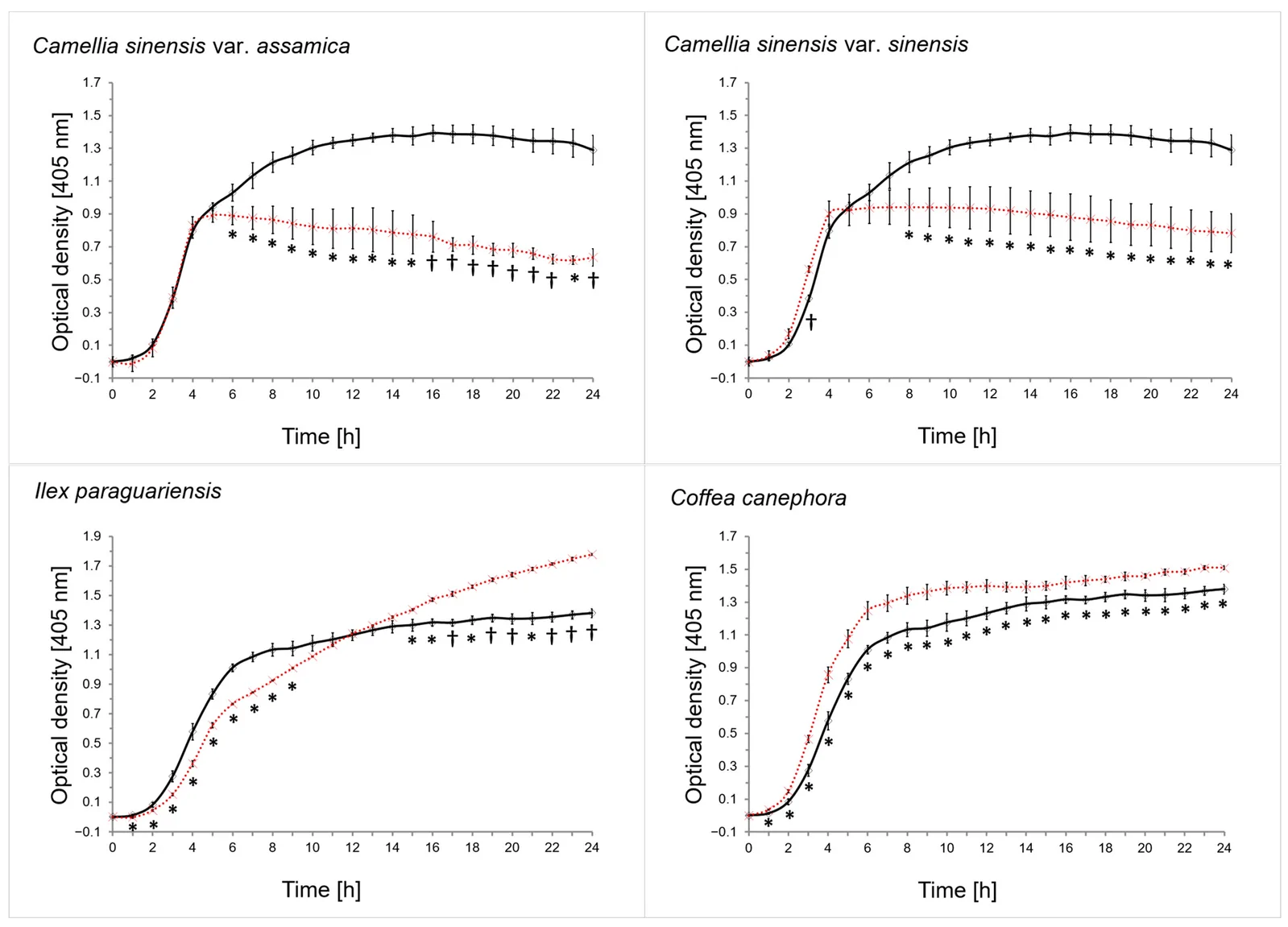
The effect of *Camellia sinensis* varieties on the growth kinetics of *Streptococcus bovis* ATCC 33317 and *Ilex paraguariensis* and *Coffea canephora* on the growth kinetics of *Escherichia coli* ATCC 25922. Growth control (**—**◇**—**), 512 µg/mL (···×···); * *p* < 0.05 vs. growth control (Welch’s *t*-test; unadjusted); † adjusted *p* < 0.05 (Bonferroni correction). Data are mean ± standard deviation, *n* = 3 technical replicates.

From the results of the primary screenings, none of the extracts from the six stimulant beverages exhibited in vitro growth-inhibitory effect against any of the six representatives of both aerobic and anaerobic intestinal cancer-related bacteria (MICs > 512 µg/mL). Out of the two drugs used as positive antibiotic controls, ciprofloxacin and tetracycline inhibited the growth of all strains of MICs in the ranges from 0.015 to 8 and from 0.125 to 8 µg/mL, respectively. The list of MICs can be found in Table 1. Measuring the effect of the extracts from the stimulant beverages on the growth kinetics of cancer-related bacteria helped to identify certain patterns of growth-modulatory activities that would not have been discovered by the standard MIC assessment. None of the extracts affected the growth of *B. fragilis*. The degree of modulatory/stimulatory effect of individual concentrations of the tested extracts after the measured period of bacterial growth kinetics can also be found in Table 1. For the extracts that notably affected the length of the lag phase, the calculated time differences are presented in Table 2. Figure 1, Figure 2, Figure 3 and Figure 4 present selected representative growth curves illustrating notable changes in growth dynamics, including growth modulation/stimulation and/or alterations in the lag phase.

Regarding *C. septicum*, the modulatory effects were mainly observed with *I. paraguariensis*. It considerably prolonged the lag phase (94–128 min) at the concentrations 128–512 µg/mL and simultaneously reduced the growth to 56% at the concentration 512 µg/mL. Then, the lag phase of this bacterium was extended by 123 and 61 min in the presence of the respective extracts from *C. canephora* and *C. sinensis var. assamica* at the concentration 512 µg/mL. In the cases of the latter extract, the growth was at the same time reduced to 41% of the growth control. The range of three concentrations of *A. linearis* (128–512 µg/mL) reduced the growth rate of *C. septicum* resulting in reduction in the growth to 66–48%. Effect of the prime four mentioned extracts on growth kinetics of *C. septicum* is displayed in Figure 1. Regarding *P. anaerobius*, mainly the growth rate was affected resulting in reduced turbidity in the presence of some of the extracts (Figure 2). Specifically, three concentrations of *A. linearis* (128–512 µg/mL), two of *C. sinensis* var. *sinensis* (256–512 µg/mL), and one of *C. sinensis* var. *assamica* and *I. paraguariensis* (512 µg/mL) modulated the growth to 63–78% of the growth control. Moreover, the lag was prolonged by 72 and 65 min in cases of *I. paraguariensis* concentrations 512 and 256 µg/mL, respectively.

Although the growth of *F. necrophorum* was affected by all extracts in a concentration-dependent manner, none of them was able to prolong the lag phase (Figure 3). Specifically, *C. sinensis* var. *assamica*, *I. paraguariensis*, and *A. linearis* modulated this bacterium at the concentrations 512 and 256 µg/mL, leading to a reduction in the final growth (26–34% and 48–70% of control, respectively). In the case of the concentration 512 µg/mL, there was no virtual stationary phase, and after 5 h the growth appeared to transition into the death phase. *I. paraguariensis*, and both tea varieties notably affected the bacterium even at the concentration 128 µg/mL, causing the drop of turbidity to 31–57% of the control after 6 h. Similarly, *C. sinensis* var. *sinensis* and both species of coffee (*C. arabica* and *C. canephora*) exhibited growth reduction (41–90% of control) at the concentrations tested (64–512 µg/mL). *S. bovis* was affected in terms of the suppression of the growth comparing to the control, particularly by the concentration 512 µg/mL of both tea varieties (49–60%). The calculations also showed that the lag phase was slightly shortened by *C. sinensis* var. *sinensis* (5–19 min). In contrast, several treatments increased the relative growth of *E. coli* above the growth control. In particular, *I. paraguariensis* and *Coffea* spp. at 512 µg/mL resulted in relative growth ranging from 109 to 128%. While *I. paraguariensis* (512 µg/mL) prolonged the lag phase of this bacterium by up to 21 min, *C. canephora* (256–512 µg/mL) expedited the initiation of exponential phase by at least 18 min. Nevertheless, these small lag-time differences observed in both *E. coli* and *S. bovis* should be interpreted cautiously because they are based on technical replicates from a single experimental run. This uncertainty is particularly evident for *I. paraguariensis*-treated *E. coli*, where the 21 min difference from the growth control was small relative to the large variability among treated replicates (121 ± 53 min vs. 100 ± 1 min). The effects of the extracts affecting growth kinetics of *S. bovis* and *E. coli* are displayed in Figure 4.

## 3. Discussion

The interface between food and medicine, with particular focus on the role of diet in gastrointestinal health promotion and disease prevention, has been increasingly discussed. Unlike antibiotic drugs, dietary bioactive compounds from herbal infusions are not expected to exert strong, immediate growth-inhibitory effects and their biological relevance may instead lie in more moderate, species-specific changes in bacterial growth dynamics [61]. To the best of our knowledge, this is the first study reporting the effect of the major stimulant beverage hot-water extracts on the growth kinetics of colorectal cancer-related bacterial species *B. fragilis*, *C. septicum*, *S. bovis*, *F. necrophorum* and *P. anaerobius*. Our findings broadly accord with the generally moderate or weak in vitro antimicrobial activity reported for stimulant-beverage extracts relative to antibiotics. The MICs obtained for ciprofloxacin and tetracycline were generally consistent with published susceptibility data for these bacterial groups, although some strain-level variation was observed, particularly among anaerobes [62,63]. Both *C. sinensis* extracts produced considerable modulation of growth kinetics of most of the bacteria tested. Considering *E. coli*, studies of Su et al. [47] and Goswami et al. [64] detected MICs of the *C. sinensis* var. *sinensis* and *C. sinensis* var. *assamica* extracts against the same strain of *E. coli* at 800 and 3680 µg/mL, respectively. This may be compatible with our findings, as little or no inhibition was observed at lower concentrations, whereas some modulatory effects began to emerge at 512 µg/mL. On the other hand, the study of Alghamdi [45] reported significant antimicrobial activities of *C. sinensis* var. *sinensis* water extract at notably lower concentrations (MIC = 12.5 µg/mL). However, the extraction conditions differed substantially from those used in our study, as the extract was prepared at a concentration nearly six times higher and over an extraction period of two days. This discrepancy is therefore likely attributable to differences in extract preparation. In our study, the ratio of dry material to liquid volume and the extraction time were selected to replicate common beverage preparation. Previous research has shown that varying the preparation of green tea affects the yield of biologically active compounds [65]. It is well known that the main biologically active constituents of beverages prepared from *C. sinensis* are polyphenols, particularly catechins and theaflavins in unoxidized and oxidized teas, respectively [66]. Although EGCG is one of the most studied compounds from the point of view of its antimicrobial properties, the previously reported inhibitory activity against intestinal bacteria is moderate or weak. For example, the MICs defined for EGCG were 500–2500 µg/mL against *F. nucleatum* [67,68], 62.5–250 µg/mL against *V. cholerae* isolates [67], 32 µg/mL against *Campylobacter jejuni* [69], and 250–500 µg/mL against *Clostridium perfringens* [70]. In the study of Lee et al. [71], EGCG only slightly affected the growth kinetics of *E. coli* O157:H7 at the concentration 100 µg/mL. Although not directly comparable because the tested proanthocyanidins were derived from *Dorycnium rectum* rather than tea, Sivakumaran et al. [72] reported that material in which EGCG was the predominant extender unit inhibited the growth of *P. anaerobius* even at the concentration of 100 µg/mL. Considering that antibacterial activity of tea catechins results from a variety of mechanisms, mainly (1) inhibition of virulence factors, (2) cell wall and cell membrane disruption, (3) inhibition of intracellular enzymes, (4) oxidative stress, DNA damage, and (5) iron chelation [73], it is expected that their synergistic effect will be crucial to produce inhibitory effects even in the low quantities. The same as EGCG, theaflavins produced activity against *F. nucleatum* at MIC of 500 µg/mL [68]. In the study of Mohammadi et al. [70], they were comparably more active against *C. perfringens* (MICs = 125–500 µg/mL). Although clinical evidence for a direct preventive effect of tea on colorectal cancer remains limited and inconsistent, available human and in vivo studies indicate that tea can modulate intestinal bacterial groups associated with colorectal carcinogenesis, providing relevant biological context for the present in vitro findings. For example, a clinical trial of Yuan et al. [43] showed that consumption of green tea elevates the *Bifidobacterium*-to-Enterobacteriaceae ratio and decreases the proinflammatory *Fusobacterium* genus. In the study of Ou et al. [74], it was also found that tea polyphenols can inhibit the activity of matrix metalloproteinase 7 upregulated by *F. nucleatum* that is responsible for carcinogenesis.

*I. paraguariensis* is another beverage whose hot-water extract exhibited modulation of the growth kinetics of several oncogenic bacteria tested. While this was characterized mainly by growth suppression, in the case of *E. coli* the growth was slightly stimulated by the extract despite a partial delay in the lag phase. The available literature on activities of this plant against *E. coli* is not particularly consistent with these findings. First, Burris et al. [52] reported MICs ranging from 150 to 800 µg/mL. In this case, the discrepancy could be explained by the use of a different bacterial strain (ATCC 43894), the dialyzed aqueous extracts, and the lower microbial counts in the inoculum. In the experiment of Paluch et al. [75], another strain of *E. coli* (ATCC 10536) was studied in terms of the modulations of growth kinetics in the presence of the *I. paraguariensis* water extract. The concentration of 1000 µg/mL was able to slow and inhibit bacterial growth by approximately 50%. It can be hypothesized that the slightly higher final turbidity observed at 512 µg/mL in our study may represent part of a hormetic concentration–response relationship, in which bioactive compounds produce modest stimulation at sub-inhibitory concentrations but inhibit bacterial growth once the concentration exceeds the inhibitory threshold [76]. Previous studies reported activities of this plant against several intestinal pathogens including *Salmonella* spp. (MICs = 780–6250 µg/mL) [53,54], enterococci (MICs = 400–1870 µg/mL, >30,000 µg/mL) [55,56], and *L. monocytogenes* (MIC = 3130 µg/mL) [54]. Paluch et al. [75] attributed the antimicrobial activity of *Ilex* spp. to the synergistic effect of diverse saponins and polyphenols. As claimed in the study, saponins may increase the activity of polyphenols, such as hydroxycinnamic acid derivatives and flavonoids, or directly disrupt bacterial cell membranes. As in the case of *C. sinensis*, the positive impact of *I. paraguariensis* on gut microbiota with relevance to some oncogenic bacteria has previously been supported by more than in vitro tests. For example, in the study of Olate-Briones et al. [44], the administration of yerba mate reduced the *E. rectale*, *C. coccoides*, Enterobacteriaceae and increased the *Lactobacillus*/*Lactococcus* groups in the gut microbiota of mice.

The modest reduction in *E. coli* growth observed with *A. linearis* in our study indicates a concentration-dependent modulatory potential that could become more pronounced at concentrations above those tested. Correspondingly, this may reflect a concentration-dependent effect, as a previous study reported rooibos aqueous extract inhibition of the identical strain at a higher concentration (MIC = 1500 µg/mL), although, at significantly higher concentrations, it was also able to inhibit the growth of other intestinal bacteria, namely *E. faecalis* (MIC = 3 mg/mL) and *B. cereus* (MIC ≥ 8 mg/mL) [57]. Unlike the tea made from *C. sinensis*, antimicrobial properties of *A. linearis* are not well recognized. Its main bioactive constituents are polyphenols and phenolic acids, such as aspalathin, nothofagin, rutin, and ferulic acid [77,78], whereas the antioxidant, antimutagenic, and hypoglycemic properties have particularly been highlighted [79]. Although previous study showed that aspalathin-rich *A. linearis* extract can inhibit activity against some foodborne pathogens (*S. aureus*, *Listeria monocytogens*, and *E. coli*), the active concentrations were very high (5 × 10^6^ and 1 × 10^7^ µg/mL). Then, the study of Simpson et al. [80] showed that polyphenol-rich fermented *A. linearis* extract can inhibit the growth of *Staphylococcus epidermis* and *S. aureus* but not of *E. coli*. However, with an identical test using some of the above-named compounds, no inhibition was observed. The suggested reasons by the author for such discrepancy were as follows: bacterial uptake of polyphenols, the unidentified polyphenols/unconsidered compounds present in the extracts, and reduced molecular efficacy. Therefore, future studies should aim to clarify these ambiguities. Considering the coffee extracts, *C. canephora* was previously tested in vitro against *F. nucleatum* but showed no effect even at extremely high concentrations (MIC > 200 mg/mL) [81]. Generally, the literature indicates that antimicrobial activity of both *C. arabica* and *C. canephora* is rather weak. For example, the MICs determined previously were as follows: 4–24.9 mg/mL (*S. aureus*), 6.25–25 mg/mL (*V. chloreae*), and 12.4 mg/mL (*S. mutans*) [48,49,50]. Antonio et al. [82] identified bacteriostatic activity of the coffee extract against *S. mutans* and suggested that the overall antibacterial effect may result from the combined action of chlorogenic acid, trigonelline and caffeine. In the same study, *C. canephora* showed better antimicrobial performance than *C. arabica*. A similar species-related tendency was observed in our study for some bacteria, particularly *F. necrophorum*, against which *C. canephora* produced stronger growth suppression than *C. arabica*. Previous experiments with human fecal microbiota treated with coffee samples showed that those with the highest levels of chlorogenic acid induced selective modulation of individual taxa. However, the study did not provide clear evidence that coffee samples and chlorogenic acid suppress some microbial taxa associated with inflammation in colorectal carcinogenesis. In contrast; increases were observed in the *C. coccoides*–*E. rectale* group [83,84].

Several limitations of the present study should be acknowledged. Each bacterial species was represented by a single reference strain; therefore, the findings cannot capture strain-level variation in colorectal cancer-associated virulence traits and should not be generalized to diverse clinical isolates or specific oncogenic pathotypes, such as colibactin-producing *E. coli* [85]. Moreover, the six tested beverage extracts were not chemically profiled. The suggested phytochemicals remain plausible but unconfirmed contributors to the observed effects, and differences from studies using other extraction procedures cannot be explained by extract composition without measuring it. Future studies should measure total phenolic content in each extract, assess flavonoids separately where relevant, and quantify beverage-specific marker compounds using liquid chromatographic methods of beverage-specific marker compounds to enable composition–activity comparisons [86]. Tests of individual compounds, active fractions or reconstituted mixtures would then be needed to establish which constituents contribute to growth modulation. Whereas the MIC assays were conducted in three independent experiments, growth kinetics were assessed in a single run with three technical replicates. Repeating the kinetic assays in three independent experiments would provide stronger evidence that the observed effects are reproducible [87]. As an additional methodological refinement, viable-cell counts at selected time points could test whether changes in optical density correspond to changes in bacterial viability [88]. Finally, isolated cultures do not reproduce intestinal microbial interactions or metabolism of beverage constituents. Testing multiple virulence-characterized isolates and mixed microbial communities at plausible intestinal exposures should precede in vivo studies and any inference about colorectal cancer prevention [89].

## 4. Materials and Methods

### 4.1. Plant Materials

Three samples of commercially available *C. sinensis* var. *assamica*, *C. sinensis* var. *sinensis*, *A. linearis*, *C. arabica* and *C. canephora* were purchased from Herbs Life (Sokolov, Czechia) and Manu Cafe (Ludgerovice, Czechia), whereas *I. paraguariensis* was purchased from Salvia Paradise (Zajecov, Czechia). The coffee samples were purchased as roasted whole beans, the tea samples as dry rolled leaves, and rooibos and yerba mate as dry chopped leaves. The beverages were prepared as hot-water infusions to approximate their conventional mode of consumption, consistent with the treatment of tea, yerba mate and coffee beverages [90]. All samples were ground into a fine powder using an electric mill GM 100 (Retsch, Haan, Germany) and used for the extraction. Four grams of the powder were poured over 200 mL of boiling distilled water and left leaching for 5 min. During the leaching process the samples were stirred using the laboratory magnetic stirrer (RH basic 2, IKA, Staufen, Germany) and afterwards poured through a sieve into a volumetric beaker. After the infusions had completely cooled, they were filtered through the filter paper with a grammage of 84 g/m^2^ (BOECO, Hamburg, Germany). Twenty milliliters of homogenized liquids were lyophilized by a freeze-dryer (Coolsafe, Labogene, Lillerød, Denmark) for 75 h. Extract yield was calculated as the percentage ratio of the dry mass of the lyophilized extract to the initial mass of the plant material. The differences in extract yield likely reflect variation in the amount of water-extractable material among the plant products. Dried residues of the resulting freeze-dried hot-water extracts were completely dissolved in 100% dimethyl sulfoxide (DMSO) (Penta, Prague, Czechia), used solely as the vehicle for reconstitution, to obtain a stock solution of the final concentration 51.2 mg/mL and stored at −20 °C until use. The list of plant materials of all stimulant beverages tested can be found in Table 3.

### 4.2. Bacterial Strains and Media

The antibacterial activity was determined against 6 representatives of both Gram-positive and Gram-negative and aerobic/anaerobic colorectal cancer-related bacteria. Standard American Type Culture Collection (ATCC) and Czech Collection of Microorganisms (CCM) strains, namely, *B. fragilis* ATCC 25285, *C. septicum* ATCC 12464, *E. coli* ATCC 25922, *S. bovis* ATCC 33317, *F. necrophorum* CCM 5981 and *P. anaerobius* CCM 3790, were obtained from Oxoid (Basingstoke, United Kingdom) and CCM (Brno, Czechia) and stored at 3–5 °C in culti-loops. Mueller–Hinton broth (MHB) was used as the maintenance and growth medium for *E. coli*, Wilkins–Chalgren broth (WCB) supplemented with 5 g/L soya peptone and 0.5 g/L cysteine was used for *C. septicum*, *F. necrophorum* and *P. anaerobius*, whereas Brain Heart Infusion (BHI) broth was used for *B. fragilis* and *S. bovis*. All media were purchased from Oxoid (Basingstoke, United Kingdom). All medium components, including the required supplements, were added prior to autoclaving. The pH was adjusted to 7.6, and the complete media were sterilized using a steam sterilizer (Tuttnauer 3870ELV, Netherlands).

### 4.3. Determination of the MICs

For the effective assessment of in vitro antimicrobial activity, a broth microdilution method using 96-well microtiter plates was used according to the Clinical and Laboratory Standards Institute (CLSI) [91] guidelines modified by Cos et al. [92], where MICs [µg/mL] were assessed. Before the testing, aerobic bacteria (*E. coli* and *S. bovis*) were sub-cultured in the relevant media at 37 °C for 24 h, whereas the anaerobic strains (*C. septicum*, *F. necrophorum*, and *P. anaerobius*) were cultured at 37 °C for 48 h inside the Whitley A35 Anaerobic Workstation (Don Whitley Scientific, Bingley, UK). The standard anaerobic gas mixture of 10% H_2_, 10% CO_2_, and 80% N_2_ (Linde Gas, Prague, Czechia) was used to create the anaerobic conditions. All 6 samples (freeze-dried residues) of beverage hot-water extracts dissolved in DMSO were 2-fold diluted in appropriate growth media (range from 64 to 512 µg/mL) using the Freedom EVO 100 automated pipetting platform (Tecan, Mannedorf, Switzerland). The highest concentration was prepared at a 1:99 (*v*/*v*) ratio of DMSO stock solution to growth medium. Manual pipetting with a multichannel pipette (Eppendorf, Hamburg, Germany) was used for the experiments prepared inside the anaerobic station. For each bacterial strain, growth controls containing the corresponding growth medium and inoculum without extract, sterility controls containing medium only, and blank controls containing the tested extract without bacterial inoculum were included. The overnight cultures were directly diluted in fresh corresponding growth medium in a sterile tube to achieve an inoculum density of 1.5 × 10^8^ Colony-Forming Units/mL corresponding to the 0.5 McFarland standard, as measured using Densi-La-Meter II (Lachema, Brno, Czechia). The cultures were then inoculated in the plates (5 µL/well). The final assay volume in each well was 100 µL. Incubation was carried out under the same conditions as in the pre-culture step, whereas the extracts and bacterial strains remained in contact under static conditions throughout that period. Following incubation, bacterial growth was first evaluated visually in accordance with the CLSI broth microdilution guidelines. The optical density of each well was measured at 405 nm (OD_405 nm_) after orbital shaking of microtiter plates using a Cytation 3 Imaging Reader (BioTek, Winooski, VT, USA) before and after the growth period. The lowest concentration (µg/mL) of the extracts that inhibited the bacterial growth by ≥90% was defined as MIC. Every test consisted of three independent experiments, each conducted in triplicate. The final MIC value was calculated as mode or median depending on if the triplicate endpoints were within the two- or three-dilution ranges. Ciprofloxacin and tetracycline (Sigma-Aldrich, Prague, Czechia) were tested as positive antibiotic controls. Before the dilution in the growth medium, the former was dissolved in DMSO (Sigma-Aldrich, Prague, Czechia) and the latter in ethanol (Lach-Ner, Neratovice, Czechia). As a result of the control experiments performed in this study without dissolved extracts and compounds, none of the used solvents inhibited bacterial growth of any strain at the tested concentrations (≤1%) [93].

### 4.4. Monitoring Growth Kinetics

For monitoring of bacterial growth kinetics, the protocol of the broth microdilution method described above was used with the following modifications. Beverage hot-water extract samples were 2-fold diluted in MHB (for *E. coli*), BHI (for *S. bovis*), and WCB (for *C. septicum*, *F. necrophorum* and *P. anaerobius*) in ranges of 64–512 µg/mL. During 24 h of incubation, absorbance measurements in combination with the regular orbital shaking were performed every hour by Cytation 3 Imaging Reader (BioTek, VT, USA). The plates inoculated with anaerobes were prepared and incubated in the Whitley A35 Anaerobic Workstation (Don Whitley Scientific, Bingley, United Kingdom) and manually withdrawn every hour for each absorbance measurement. Due to differences in the onset of bacterial growth and the time required to reach maximum observed optical density or the stationary phase, the monitoring period for individual anaerobic strains was adjusted accordingly. To avoid oxygen contamination that could negatively affect the growth of the anaerobic bacteria, a unique plate was prepared for each measurement. Therefore, minor fluctuations between consecutive absorbance measurements may reflect inter-plate variability, particularly at low bacterial densities. The growth-kinetics assay was performed in a single experimental run with three technical replicate wells for each treatment and control condition. Accordingly, the triplicates represent within-experiment technical variability rather than independent experimental replicates.

### 4.5. Calculations and Statistics

The lag time was defined as the time at which the extrapolated slope of the exponential phase intercepts a horizontal line, extrapolated from the starting inoculum concentration. The length of the lag phase of the aerobic bacteria alone or in the presence of various extract concentrations was calculated by Gen5 Image+ 3.11 software (BioTek, VT, USA), which determines lag time from the relationship between the line of maximum slope during the propagation phase and the initial absorbance baseline. In the case of the anaerobic strains, the lag phase was calculated in Microsoft Excel 365, Version 2609 (Microsoft corporation, WA, USA) based on established approaches for the mathematical determination of bacterial lag phase from growth curves, using backward extrapolation of the fitted exponential-growth region toward the initial absorbance baseline [94,95,96]. Although different calculation procedures were used for aerobic and anaerobic strains, both approaches estimate lag time by extrapolating the exponential-growth region toward the initial absorbance baseline, thus following the same general kinetic principle. For each growth curve, the complete measurement series was first inspected and a curve-specific range corresponding to the clear, sustained exponential-growth phase was selected from the overall measurement period. An exponential trendline was fitted to the selected range according to the following equation:

*y* = *a e*^{*bx*}^

where *y* is the optical density predicted by the fitted exponential growth curve, *x* is time (h), *a* is the coefficient of the fitted exponential trendline, *b* is the exponential growth-rate constant (h^−1^), and *e* is Euler’s number.

The exponential trendline was subsequently used to estimate the lag-phase duration according to:

$$t_{\mathrm{lag}}=\frac{\ln\left(\frac{y}{a}\right)}{b}\times 60$$

where *t_lag_* is the lag phase time, *y* is the optical density value at the time of the start of exponential growth, *a* is the initial optical density value, and *b* is the growth-rate constant. The multiplication by 60 converts the calculated time from hours to minutes.

For extracts showing the notable changes in growth dynamics, including growth modulation and prolongation or shortening of the calculated lag phase, growth curves plotting the time dependence of optical density at wavelength of 405 nm were constructed using Microsoft Excel 365, Version 2609 (Microsoft corporation, WA, USA) and selected for presentation in the figures. Similar to the MIC determination, relative growth was calculated for each concentration of all tested extracts at the end of the monitored growth period and expressed as a percentage of the growth control using the following formula:

$$\mathrm{Relative growth}\left(\%\right)=\frac{OD_{\mathrm{ext}}}{OD_{\mathrm{gc}}}\times 100$$

where *OD_ext_* is the optical density measured in the presence of individual concentrations of the extracts and *OD_gc_* is the optical density measured in the presence of the microorganism only.

For each growth kinetics curve, the mean optical density of each extract concentration was also compared with the corresponding growth control at each measurement time using Welch’s *t*-test based on the mean, standard deviation, and three replicate measurements [97,98]. Raw *p* values were initially evaluated at *p* < 0.05. Because multiple time points were tested within each growth curve, *p* values were additionally adjusted using the Bonferroni correction to account for multiple comparisons and reduce the probability of false-positive results [99,100]. As the three measurements represented technical replicates within a single experimental run rather than independent experiments, these statistical comparisons were used as exploratory within-run analyses. Both raw and Bonferroni-adjusted significance were indicated in the growth-kinetics graphs. Although Bonferroni correction controls for multiple time-point comparisons, it should be noted that it does not explicitly account for temporal autocorrelation between successive measurements within the same growth curve. For comparisons of lag time, the same exploratory tests were likewise applied to the replicate lag-time estimates for each concentration versus the corresponding growth control.

Welch’s *t*-test:

$$t=\frac{\left|M_{\mathrm{GC}}-M_{T}\right|}{\sqrt{\left(\frac{SD_{\mathrm{GC}}^{2}}{n_{\mathrm{GC}}}+\frac{SD_{T}^{2}}{n_{T}}\right)}}$$

where *M_GC_* is the mean optical density of the growth control, *M_T_* is the mean optical density of the treatment, *SD_GC_* and *SD_T_* are their respective standard deviations, and *n_GC_* and *n_T_* are the numbers of replicate measurements (*n* = 3).

Bonferroni correction:

*p_adjusted_* = min(*p_raw_* × *N*,1)

where *p*_raw_ is the *p* value obtained from Welch’s *t*-test, *N* is the number of time-point comparisons within the respective growth curve, and *p_adjusted_* is the Bonferroni-adjusted *p* value. A threshold of *p* < 0.05 was used for the exploratory comparisons.

## 5. Conclusions

In summary, this study showed that stimulant beverage hot-water extracts, especially *C. sinensis* and *I. paraguariensis*, produce in vitro activities on colorectal cancer-related bacteria by modulating their growth kinetics. More occasional and less pronounced concentration-dependent activities were also detected in the cases of *A. linearis*, *C. arabica*, and *C. canephora.* The tested beverage extracts were able to reduce the growth and/or prolong the lag phase of several strains, especially *C. septicum*, *F. necrophorum*, *P. anaerobius*, and *S. bovis*, whereas *B. fragilis* was unaffected and slight stimulatory effects were observed for *E. coli* under some conditions. Further studies should confirm these findings using multiple virulence-characterized isolates, independently replicated growth-kinetics experiments, chemically characterized extracts, and more complex microbial models that better reflect intestinal interactions and metabolism of beverage constituents before their relevance to colorectal cancer can be established.

## Figures and Tables

**Table 1:** Modulatory effects of hot-water extracts from stimulant beverages on colorectal cancer-related bacteria.

Bacterial Strain/Plant Species	MIC (µg/mL)	Extract Concentration (µg/mL)/Relative Growth (%)			
		64	128	256	512
*Clostridium septicum* ^b^					
*A. linearis*	>512	93 ± 12.0	66 ± 16.3 *	59 ± 29.0	48 ± 5.1 ^†^
*C. sinensis var. sinensis*	>512	95 ± 6.4	93 ± 9.7	72 ± 6.2 ^†^	63 ± 10.9 ^†^
*C. sinensis var. assamica*	>512	97 ± 3.6	87 ± 6.1	84 ± 6.8	41 ± 18.2 *
*C. arabica*	>512	92 ± 4.1	100 ± 7.3	85 ± 9.0	74 ± 19.5
*C. canephora*	>512	96 ± 19.6	108 ± 7.2	105 ± 2.6	82 ± 10.0
*I. paraguariensis*	>512	92 ± 7.9	85 ± 9.9	90 ± 3.1	56 ± 5.9 ^†^
Ciprofloxacin	0.5	- ^d^	-	-	-
Tetracycline	0.125	-	-	-	-
*Escherichia coli* ^c^					
*A. linearis*	>512	104 ± 1.3	107 ± 0.8	99 ± 2.9	94 ± 6.7
*C. sinensis var. sinensis*	>512	105 ± 0.2	118 ± 3.0 *	115 ± 1.8 *	74 ± 8.6 *
*C. sinensis var. assamica*	>512	104 ± 0.6	107 ± 2.8	109 ± 0.9	85 ± 3.8 *
*C. arabica*	>512	107 ± 1.3	125 ± 0.6 *	128 ± 0.7 *	116 ± 3.0 *
*C. canephora*	>512	104 ± 1.1	110 ± 4.4	112 ± 2.1	109 ± 4.8
*I. paraguariensis*	>512	109 ± 4.4	112 ± 1.1	127 ± 2.0 *	128 ± 5.7 ^†^
Ciprofloxacin	0.015	-	-	-	-
Tetracycline	2	-	-	-	-
*Fusobacterium necrophorum* ^a^					
*A. linearis*	>512	84 ± 8.0	82 ± 5.8	49 ± 14.1 *	34 ± 24.4 *
*C. sinensis var. sinensis*	>512	90 ± 8.9	56 ± 7.4 *	48 ± 15.7 *	47 ± 8.7 *
*C. sinensis var. assamica*	>512	80 ± 10.3	57 ± 9.7 *	70 ± 14.1	26 ± 9.8 ^†^
*C. arabica*	>512	79 ± 13.3	95 ± 26.6	56 ± 10.6 *	73 ± 24.0
*C. canephora*	>512	72 ± 9.6	67 ± 10.6 *	57 ± 16.6 *	41 ± 9.9 ^†^
*I. paraguariensis*	>512	80 ± 8.7	31 ± 8.6 ^†^	48 ± 10.4 ^†^	27 ± 21.0 ^†^
Ciprofloxacin	1	-	-	-	-
Tetracycline	8	-	-	-	-
*Peptostreptococcus anaerobius* ^b^					
*A. linearis*	>512	96 ± 12.2	74 ± 17.4	75 ± 31.0	73 ± 5.5 *
*C. sinensis var. sinensis*	>512	98 ± 6.7	97 ± 10.4	78 ± 6.6 *	73 ± 11.7 *
*C. sinensis var. assamica*	>512	99 ± 4.7	96 ± 6.5	111 ± 7.3	63 ± 19.5
*C. arabica*	>512	95 ± 4.3	103 ± 7.8	89 ± 9.6	80 ± 20.8
*C. canephora*	>512	98 ± 9.0	111 ± 7.7	112 ± 2.8	86 ± 10.7
*I. paraguariensis*	>512	94 ± 7.9	89 ± 10.6	97 ± 3.3	64 ± 6.3 ^†^
Ciprofloxacin	0.5	-	-	-	-
Tetracycline	8	-	-	-	-
*Streptococcus bovis* ^c^					
*A. linearis*	>512	98 ± 6.5	94 ± 10.7	91 ± 5.1	73 ± 2.7 ^†^
*C. sinensis var. sinensis*	>512	106 ± 2.7 *	104 ± 5.3	101 ± 5.2	60 ± 1.8 ^†^
*C. sinensis var. assamica*	>512	98 ± 6.8	92 ± 7.4	83 ± 4.0 ^†^	49 ± 4.4 ^†^
*C. arabica*	>512	72 ± 1.5 ^†^	98 ± 2.9	90 ± 5.7	81 ± 1.6 ^†^
*C. canephora*	>512	71 ± 2.1 ^†^	92 ± 3.0 *	87 ± 1.1 ^†^	77 ± 0.9 ^†^
*I. paraguariensis*	>512	73 ± 4.8 ^†^	88 ± 2.0 ^†^	79 ± 1.5 ^†^	54 ± 0.4 ^†^
Ciprofloxacin	8	-	-	-	-
Tetracycline	8	-	-	-	-

MIC: Minimum inhibitory concentration (data are mode/median, *n* = 3 independent experimental replicates); modulatory effect measured after ^a^ 7 h, ^b^ 14 h, and ^c^ 24 h; ^d^ not applicable; * *p* < 0.05 vs. growth control (Welch’s *t*-test; unadjusted); ^†^ adjusted *p* < 0.05 (Bonferroni correction). Data are mean ± standard deviation, *n* = 3 technical replicates.

**Table 2:** The effect of hot-water extracts from stimulant beverages on the lag phase parameters of colorectal cancer-related bacteria.

Bacterial Strain/Plant Species	Concentration (µg/mL)								
	Lag Time Duration (min)					Time Difference (min) ^a^			
	0 (Growth control)	64	128	256	512	64	128	256	512
*Clostridium septicum*									
*C. canephora*	487 ± 5	480 ± 3	479 ± 18	474 ± 13	609 ± 8 ^†^	−6	−7	−12	123
*C. sinensis* var. *assamica*	487 ± 5	555 ± 0 ^†^	548 ± 9 ^†^	524 ± 7 ^†^	548 ± 46	69	62	37	61
*I. paraguariensis*	487 ± 5	580 ± 3 ^†^	585 ± 14 ^†^	590 ± 7 ^†^	615 ± 2 ^†^	94	99	103	128
*Peptostreptococcus anaerobius*									
*I. paraguariensis*	543 ± 10	559 ± 1	546 ± 9	608 ± 3 ^†^	615 ± 2 ^†^	15	3	65	72
*Streptococcus bovis*									
*C. sinensis var. sinensis*	75 ± 2	68 ± 0 *	70 ± 5	61 ± 3 ^†^	56 ± 9	−7	−5	−14	−19
*Escherichia coli*									
*C. canephora*	100 ± 1	104 ± 1 *	76 ± 1 ^†^	74 ± 0 ^†^	82 ± 1 ^†^	4	−24	−26	−18
*I. paraguariensis*	100 ± 1	115 ± 54	96 ± 0 *	107 ± 8	121 ± 53	15	−4	7	21

^a^ Lag time of extract—lag time of growth control; * *p* < 0.05 vs. growth control (Welch’s *t*-test; unadjusted); ^†^ adjusted *p* < 0.05 (Bonferroni correction). Data are mean ± standard deviation, *n* = 3 technical replicates.

**Table 3:** Plant materials of stimulant beverages.

Plant Species	Common Name	Supplier	Tested Part	Extract Yield (%)
*Aspalathus linearis*	Rooibos	Herbs Life (Sokolov, Czechia)	Leaves	19.3
*Coffea arabica*	Arabica coffee	Manu Cafe (Ludgerovice, Czechia)	Seeds	24.6
*Coffea canephora*	Robusta coffee	Manu Cafe (Ludgerovice, Czechia)	Seeds	27.2
*Camellia sinensis* var. *assamica*	Black tea	Herbs Life (Sokolov, Czechia)	Leaves	21.5
*Camellia sinensis* var. *sinensis*	Green tea	Herbs Life (Sokolov, Czechia)	Leaves	22.8
*Ilex paraguariensis*	Yerba maté	Salvia Paradise (Zajecov, Czechia)	Leaves	13.6

## Data Availability

The original contributions presented in this study are included in the article. Further inquiries can be directed to the corresponding author.

## Abbreviations

The following abbreviations are used in this manuscript:

ATCC	American Type Culture Collection
BHI	Brain Heart Infusion
CCM	Czech Collection of Microorganisms
CLSI	Clinical and Laboratory Standards Institute
DMSO	Dimethyl Sulfoxide
EGCG	Epigallocatechin Gallate
MHB	Mueller–Hinton Broth
MIC	Minimum Inhibitory Concentration
WCB	Wilkins–Chalgren Broth
